# Supplementary material for: Modeling the impact of racial and ethnic disparities on COVID-19 epidemic dynamics
Source: eLife. 2021 May 18;10:e66601. doi: 10.7554/eLife.66601 (PMC8221808; doi:10.7554/eLife.66601)
Supplement: Supplementary file 1. [file elife-66601-supp1.docx]

| A | B | C | D | E |
| --- | --- | --- | --- | --- |
| A 0.616 | 0.178 | 0.101 | 0.298 | 0.284 |
| B 0.161 | 0.509 | 0.249 | 0.213 | 0.236 |
| C 0.069 | 0.188 | 0.572 | 0.078 | 0.215 |
| D 0.128 | 0.101 | 0.049 | 0.375 | 0.164 |
| E 0.026 | 0.024 | 0.029 | 0.035 | 0.102 |

Exposure index matrix for NYC. From the perspective of an individual from column *j*, each row gives the proportion of people in an average neighborhood that are from demographic group *i*. Group A denotes non-Hispanic whites, B denotes Hispanics or Latinos, C denotes non-Hispanic African Americans, D denotes non-Hispanic Asians, and E denotes multiracial or other demographic groups.

| A | B | C | D | E |
| --- | --- | --- | --- | --- |
| A 0.770 | 0.432 | 0.269 | 0.570 | 0.532 |
| B 0.119 | 0.358 | 0.278 | 0.130 | 0.189 |
| C 0.038 | 0.142 | 0.378 | 0.067 | 0.126 |
| D 0.056 | 0.047 | 0.047 | 0.208 | 0.080 |
| E 0.017 | 0.022 | 0.028 | 0.026 | 0.073 |

Exposure index matrix for Long Island. From the perspective of an individual from column *j*, each row gives the proportion of people in an average neighborhood that are from demographic group *i*. Group A denotes non-Hispanic whites, B denotes Hispanics or Latinos, C denotes non-Hispanic African Americans, D denotes non-Hispanic Asians, and E denotes multiracial or other demographic groups.

1
