# Supplementary material for: Modeling the impact of racial and ethnic disparities on COVID-19 epidemic dynamics
Source: eLife. 2021 May 18;10:e66601. doi: 10.7554/eLife.66601 (PMC8221808; doi:10.7554/eLife.66601)
Supplement: Supplementary file 2. [file elife-66601-supp2.docx]

| New York City | A | B | C | D | E |
| --- | --- | --- | --- | --- | --- |
| Proportionate mixing | 1.00 | 2.25 | 1.62 | 0.86 | 1.28 |
| Census model | 1.00 | 1.62 | 1.35 | 0.90 | 1.17 |
| Long Island | A | B | C | D | E |
| Proportionate mixing | 1.00 | 4.31 | 1.96 | 0.92 | 2.48 |
| Census model | 1.00 | 2.60 | 1.63 | 0.93 | 1.90 |

Total contact rate ratios relative to non-Hispanic whites for proportionate mixing and census models fit to New York City (top) and Long Island (bottom) data. Group A denotes non-Hispanic whites, B denotes Hispanics or Latinos, C denotes non-Hispanic African Americans, D denotes non-Hispanic Asians, and E denotes multiracial or other demographic groups.

1
