# Supplementary material for: Modeling the impact of racial and ethnic disparities on COVID-19 epidemic dynamics
Source: eLife. 2021 May 18;10:e66601. doi: 10.7554/eLife.66601 (PMC8221808; doi:10.7554/eLife.66601)
Supplement: Supplementary file 3. [file elife-66601-supp3.docx]

| Iteration | *ϵ* | A (reference) | B | C | D | E |
| --- | --- | --- | --- | --- | --- | --- |
| 0 | 0.000 | 1 | 1.000 | 1.000 | 1.000 | 1.000 |
| 1 | 0.393 | 1 | 1.696 | 1.387 | 0.897 | 1.189 |
| 2 | 0.462 | 1 | 1.609 | 1.345 | 0.905 | 1.171 |
| 3 | 0.455 | 1 | 1.617 | 1.350 | 0.904 | 1.173 |
| 4 | 0.456 | 1 | 1.616 | 1.349 | 0.904 | 1.173 |
| 5 | 0.456 | 1 | 1.616 | 1.349 | 0.904 | 1.173 |

Iterative census model fitting results for New York City. A-E are activity levels relative to Group A. Group A denotes non-Hispanic whites, B denotes Hispanics or Latinos, C denotes non-Hispanic African Americans, D denotes non-Hispanic Asians, and E denotes multiracial or other demographic groups.

| Iteration | *ϵ* | A (reference) | B | C | D | E |
| --- | --- | --- | --- | --- | --- | --- |
| 0 | 0.000 | 1 | 1.000 | 1.000 | 1.000 | 1.000 |
| 1 | 0.307 | 1 | 2.906 | 1.704 | 0.929 | 2.021 |
| 2 | 0.400 | 1 | 2.546 | 1.615 | 0.934 | 1.873 |
| 3 | 0.383 | 1 | 2.609 | 1.632 | 0.933 | 1.900 |
| 4 | 0.386 | 1 | 2.598 | 1.629 | 0.933 | 1.896 |
| 5 | 0.386 | 1 | 2.598 | 1.629 | 0.933 | 1.896 |

Iterative census model fitting results for Long Island. A-E are activity levels relative to Group A. Group A denotes non-Hispanic whites, B denotes Hispanics or Latinos, C denotes non-Hispanic African Americans, D denotes non-Hispanic Asians, and E denotes multiracial or other demographic groups.

1
